# The new small-angle X-ray scattering beamline for materials research at PETRA III: SAXSMAT beamline P62

**DOI:** 10.1107/S1600577523008603

**Published:** 2023-10-20

**Authors:** S. Haas, X. Sun, A. L. C. Conceição, J. Horbach, S. Pfeffer

**Affiliations:** a Deutsches Elektronen-Synchrotron (DESY), Notkestrasse 85, Hamburg 22607, Germany; University of Tokyo, Japan

**Keywords:** SAXS, WAXS, SAXS tensor tomography, SAXS-TT, computed tomography, anomalous scattering, ASAXS, SAXS-CT

## Abstract

The SAXSMAT beamline P62 (Small-Angle X-ray Scattering beamline for Materials Research) is a new beamline at the high-energy storage ring PETRA III at DESY. This beamline is dedicated to combined small- and wide-angle X-ray scattering (SAXS/WAXS) techniques for both soft and hard condensed matter systems. It works mainly in transmission geometry. The beamline covers an energy range from 3.5 keV to 35.0 keV, which fulfills the requirements of the user community to perform anomalous scattering experiments. Mirrors are used to reduce the intensity of higher harmonics. Furthermore, the mirrors and 2D compound refracting lenses can focus the beam down to a few micrometres at the sample position. This option with the high photon flux enables also SAXS/WAXS tensor tomography experiments to be performed at this new beamline in a relatively short time. The first SAXS/WAXS pattern was collected in August 2021, while the first user experiment was carried out two months later. Since January 2022 the beamline has been in regular user operation mode. In this paper the beamline optics and the SAXS/WAXS instrument are described and two examples are briefly shown.

## Abstract 2

The SAXSMAT beamline P62 (Small-Angle X-ray Scattering beamline for Materials Research) is a new beamline at the high-energy storage ring PETRA III at DESY. This beamline is dedicated to combined small- and wide-angle X-ray scattering (SAXS/WAXS) techniques for both soft and hard condensed matter systems.

## 1. Introduction

At the PETRA III storage ring, two dedicated small-angle X-ray scattering beamlines have been successfully operational for several years. Beamline P03 focuses on grazing-incidence small-angle X-ray scattering and microfocus applications (Buffet *et al.*, 2012 ▸; Krywka *et al.*, 2012 ▸). The second beamline, P12, operated by the EMBL (European Macromolecular Biological Laboratory), is highly optimized for BioSAXS applications (Blanchet *et al.*, 2015 ▸). To complete the portfolio of SAXS-related techniques at the PETRA III facility and to relax the overbooking of the highly requested SAXS beamlines at PETRA III a new SAXS-dedicated beamline was planned, installed and commissioned over the last three years. The additional SAXS-related beamline was realized within the successfully finished PETRA III extension project (Drube *et al.*, 2016 ▸; Franz *et al.*, 2006 ▸).

The new beamline, called SAXSMAT beamline P62 (Small-Angle X-ray Scattering beamline for Materials Research), is dedicated to combined small- and wide-angle X-ray scattering (SAXS/WAXS) for soft and hard condensed matter systems mainly in transmission geometry. This beamline allows for two advanced SAXS techniques: anomalous small-angle X-ray scattering (ASAXS) (Haas *et al.*, 2010 ▸, 2013 ▸; Sztucki *et al.*, 2011 ▸; Hoell *et al.*, 2009 ▸; Tatchev, 2008 ▸) and SAXS tensor tomography (SAXS-TT) (Liebi *et al.*, 2015 ▸, 2018 ▸; Schaff *et al.*, 2015 ▸; Guizar-Sicairos *et al.*, 2020 ▸; Conceição *et al.*, 2020 ▸; Jensen *et al.*, 2011 ▸). Special sample environments for both techniques have been developed and realized at the beamline and will be explained partially in this paper. The ASAXS method implies that the X-ray energy can be tuned continuously within a few seconds with a reliable and stable measurement of the incoming photon flux and transmitted beam intensity. To have access to as many X-ray absorption-edge energies as possible the beamline optics was chosen such that the energy can be continuously tuned from 3.5 to 35.0 keV. This allows reaching at least one absorption edge for all elements with *Z* larger than 19 (potassium). The second major technique of SAXS-TT is extremely demanding on the data-taking and handling strategy. At the SAXSMAT beamline, a continuous scan (on-the-fly data taking) has been implemented using an FPGA-based controller for synchronization of the continuous motion and the data taking. With this method, a common SAXS-TT data set can easily reach more than 1 million 2D SAXS and WAXS patterns collected within a couple of hours. Depending on the sample and the information required, different imaging approaches can be implemented to generate images from 4D (2D real space + 2D reciprocal space) to 6D (3D real space + 3D reciprocal space). Furthermore, conventional time-resolved and high-throughput combined SAXS/WAXS experiments with millisecond time resolution can be conducted at the SAXSMAT beamline as well.

The first monochromatic X-ray beam in the experimental hutch was achieved in August 2021. Two months later the first regular external user experiment had been carried out successfully. Since then, the beamline started normal regular user operation with a few longer periods for further optimization of existing components and installation of missing equipment. Since January 2022 the beamline has been in full user operation mode. Within this paper, the beamline layout and performance will be discussed and finally a few preliminary examples will be shown to highlight the potential of the beamline.

## 2. Beamline overview

**Figure 1:**
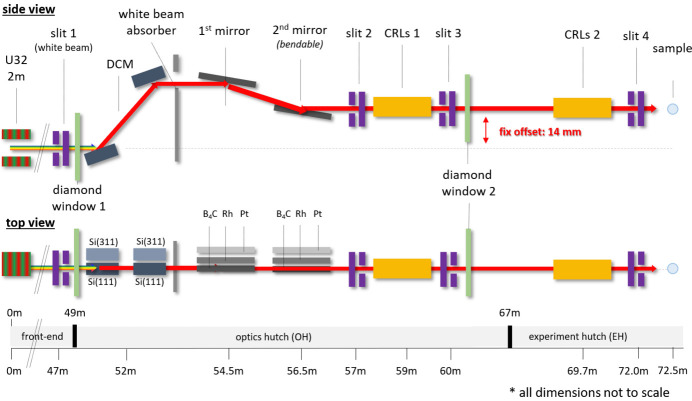
Optics beamline layout with the main components. The intensity monitors, beam position screens, valves, pumps and diamond windows are not shown.

The schematic optics beamline layout is shown in Fig. 1 ▸. The photon source of the beamline is a U32 undulator with a peak brilliance of 1.4 × 10^20^ photons s^−1^ mrad^−2^ (0.1% band­width)^−1^ at 7 keV. The monochromatization is realized by a cryo-cooled double-crystal monochromator (DCM) with Si(111) and Si(311) pairs depending on the requested energy resolution in the range 3.5–35.0 keV. For harmonic suppression, two vertical deflecting flat mirrors with B_4_C, Rh and Pt stripes are used. The incoming X-ray beam can be focused either on the sample position or on the SAXS detector by bending the second mirror or using the cylindrical stripe of the mirrors, or by using different numbers of 2D beryllium lenses. At 12 keV and 120 mA storage ring current, the beamline can reach a peak flux of ∼1.2 × 10^13^ photons s^−1^ at the sample position. The endstation of the beamline consists of a permanently installed SAXS/WAXS instrument with a flexible sample environment area. The key parameters of the beamline specification in terms of optics are given in Table 1 ▸.

### 2.1. Photon source

Table 2 ▸ summarizes the specification of the U32 undulator (Schöps *et al.*, 2016 ▸). The U32 undulator has a period length of 31.4 mm with a peak magnetic field of 0.91 T. The insertion device has 61 periods resulting in a total length of the insertion device of 2 m. The energy of the first harmonic is 2.4 keV. The total power of the U32 for this configuration is 3.8 kW with an on-axis power density of 80 W mrad^−2^. The brilliance as a function of the X-ray energy and harmonic is shown in the supporting information (Fig. S1). To fulfill the low-divergence criteria for the SAXS technique the undulator cell in the storage ring is configured as high β-mode, yielding a 140.0 µm × 5.6 µm source size and 7.9 µrad × 4.1 µrad (RMS) divergence at 10 keV photon energy. An important mode of the beamline operation will be continuous energy scans of roughly 1.5 keV range within about 1 min. That requires that the undulator gap can be continuously scanned at a certain speed. This speed depends on the starting energy and harmonic. The current design of the PETRA III extension undulator frames allows a gap scan speed of several hundred µm s^−1^, with an option to adapt the gap scan speed during the scan movement. This option allows for continuous energy scans with the undulator gap.

### 2.2. Front-end

The front-end of the beamline contains a white-beam aperture, a white-beam high-power slit system (vertical and horizontal) followed by an absorber (Schulte-Schrepping *et al.*, 2016 ▸). The absorber unit has two positions: a 300 µm-thick glassy carbon sheet and an empty position (no absorber). The last element in the front-end is the optics beam shutter. In addition, several beam position monitors are placed behind each important component in the front-end section. These beam monitors are based on a CCD (charge-coupled device) camera that looks at a diamond sheet with an etched cross in the center. Each screen can be moved in and out of the beam.

### 2.3. Beamline optics

The first optical element after the front-end section is a diamond window coupled with a CCD camera. This window separates the ultra-high-vacuum parts and acts as a beam position screen at the beginning of the optics hutch. The first active optical element is a high-heat-load cryo-cooled DCM, followed by a white-beam absorber. The next components are two vertical deflecting flat mirrors for efficient suppression of harmonics. These mirrors can also be used for focusing. Downstream of the mirrors, two slit systems have been installed with a 2D-Be lens transfocator unit in between. The optics section has been divided into several ultra-high-vacuum sections for easier maintenance work. The optics section also contains several beam position screens based on a CCD camera looking onto a diamond sheet with an etched cross in the center. These screens can be moved out of the beam during normal user operation.

#### 2.3.1. Cryo-cooled DCM

The cryo-cooled DCM of beamline P62 is the standard PETRA III high-heat-load monochromator with some modifications to further improve the monochromatic beam stability (Schulte-Schrepping *et al.*, 2013 ▸). Extensive work has been done to improve the overall stability of the monochromators for the PETRA III extension. Stability increased especially for the cryo-cooling components in the DCM vessel after these issues had been identified during systematic tests and modification at PETRA III (Sergueev *et al.*, 2016 ▸). Measures were taken by the company FMB-Oxford to improve these issues (Kristiansen *et al.*, 2015 ▸).

**Figure 2:**
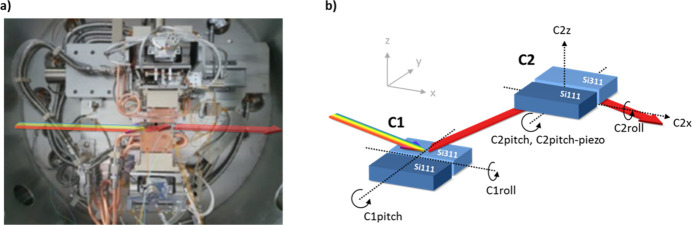
(*a*) Photograph of the PETRA III high-heat-load monochromator. (*b*) Sketch of the translation and rotation degree of freedom of the monochromator.

In Fig. 2 ▸(*a*) a photograph of the inner part of the installed DCM is shown (built by FMB-Oxford). A sketch of the fundamental principle of the DCM including the naming of the degrees of freedom is shown in Fig. 2 ▸(*b*). The monochromator is fixed-exit with an adjustable fixed beam offset in the range 21–23 mm upwards. The accessible energy range of the DCM is 3.5–35.0 keV using the Si(111) reflection. To increase the energy resolution as well as get to higher X-ray energies an additional Si(311)-pair of crystals is implemented. The fixed exit is realized by a vertical (C2z) and a horizontal (C2x) translation of the second crystal. The surface of the first crystal stays within the rotation axis and C1pitch is identical to the Bragg angle. Because both crystal cages are mounted on one backplane, the second crystal is rotated simultaneously with the first one. This means that C2pitch only affects the angle between the first and second crystal. Both pitch and roll of the second crystal can be fine-tuned separately for alignment purposes, whereas the pitch has an additional piezo-driven stage for feedback and detuning of the second crystal concerning the first crystal. A modification compared with the latest version of the PETRA III extension DCMs is the shorter longitudinal translation C2x of the second crystal by roughly 30%. This reduction can be realized due to the narrower energy range of 3.5–35.0 keV with the Si(111) reflection while keeping the fixed exit. The shorter translation allows a slightly more compact design of the inner part of the monochromator, which makes the monochromator more stable. The specifications of the travel range, repeatability and step resolution of the P62 DCM are summarized in Table 3 ▸.

#### 2.3.2. Vertical deflecting double mirror system

Two separate mirrors in vertical deflecting geometry are installed to suppress higher harmonics efficiently and keep the beam parallel to the white beam. To be able to use the mirrors over the entire energy range three flat stripes with B_4_C, Rh and Pt coatings as well as one cylindrical stripe of either B_4_C or Rh are used on both mirrors. The reflectivity curves of the double mirror system as a function of X-ray energy and incidence angle (pitch) for the different coatings are shown in the supporting information (Fig. S2). For energies between 3.5 keV and 13.0 keV, the B_4_C coating is used with a reflectivity of ∼99.8%. For the middle energy range of 12.0 keV to 21.0 keV, the Rh coating is used with a reflectivity of ∼94%. For the higher energy range from 20.0 keV to 35.0 keV, the Pt coating can be used with a reflectivity of ∼80%. An operation without the mirrors can be realized but is not the default beamline operation mode, because higher harmonic suppression is needed for high-resolution scattering experiments.

In addition to the flat stripes, the first mirror has one cylindrical B_4_C stripe and the second mirror has one cylindrical Rh stripe. The sagittal radius of both cylindrical stripes is 73 mm and 56 mm, respectively, for focusing the beam horizontally onto the sample position. The theoretical reachable horizontal beam size is of the order of 110 µm with a divergence of 23.7 µrad. Experimentally, a horizontal beam size of 125 µm at the sample position has been measured indicating a good agreement with the calculated one. The second mirror can be tangentially bent with a bending radius from 11.5 km down to 8.6 km which allows the focal length to be adjusted to match the mirror to the sample position for the different pitch angles of the mirror.

#### 2.3.3. CRL transfocator

To increase the focusing capabilities of the beamline, 2D-Be compound refractive lenses (CRLs) are installed at two positions. In the optics hutch a lens transfocator equipped with 90 beryllium lenses with a radius of 500 µm, three lenses with a radius of 1500 µm, one lens with a radius of 2500 µm and one lens with a radius of 5000 µm will be installed. The number of lenses can be varied based on the required focal length at a given X-ray energy. To have a fine adjustment of the focal length the whole transfocator can be translated in the beam direction by up to 1 m. This transfocator allows focusing either on the sample position or on the SAXS detector far away from the sample in both directions. This device is still in the commissioning phase. The estimated focused beam size at the SAXS detector position is around 5 µm × 150 µm (vertical × horizontal).

A second 2D-Be lens transfocator is installed in the experimental hutch roughly 800 mm before the sample position. This transfocator can be equipped manually with up to 30 2D-Be lenses with a radius of 50 µm. This lens system is used especially for the SAXS imaging techniques allowing a focus size of roughly 10 µm × 10 µm on the sample position. Here the round beam shape is realized by slightly defocusing the beam vertically with the mirror system to match the horizontal beam size. This system is fully commissioned and in regular use for the SAXS-tensor tomography experiments.

### 2.4. Endstation

**Figure 3:**
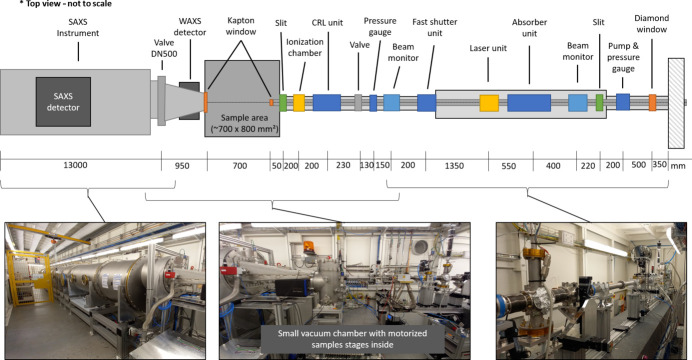
Top view sketch of the instrumentation of the experimental hutch (not to scale). For each component the distance to the next component is given in mm. In the lower part photographs of the instrumentation are shown. At the sample area a small vacuum chamber with motorized linear stages for sample translation was installed.

Figure 3 ▸ shows a top-view sketch as well as photographs of the instrumentation in the experimental hutch. The sketch is not to scale but all components and the locations are shown. The first element in the experimental hutch is again a diamond window with a CCD camera on the side. This window separates the ultra-high-vacuum (∼1 × 10^−9^ mbar) path in the optics hutch from the high-vacuum (1 × 10^−4^ mbar) path in the experimental hutch. Shortly behind the window, the first slit system from JJ X-ray is installed. The next component is a precision X-ray attenuation unit (ADC ABS-300) based on moving metal foils in and out of the beam. A wide range of attenuation across the entire energy range is realized by 11 different optimized metal foils: Al (20, 40, 80, 160, 200, 400, 800, 1600 µm), Cu (7 µm), Mo (25 µm) and W (2 mm). The last slot (2 mm W) can be used as a slow shutter absorbing all photons. The control of the unit is based on an SPS system from Beckhoff that can be easily controlled via a Tango server. Further downstream an optical mirror inside a vacuum cube can be moved into the X-ray beam position. This mirror is used to couple a green laser into the system to mimic the X-ray beam path. The laser unit is mounted outside the vacuum chamber and has five degrees of freedom for aligning the laser beam path to the X-ray beam path. Once both paths are matching only the cube with the mirror inside must be moved 10 mm to the side and the X-ray beam can pass the unit without interaction with the laser setup. The next component is a fast-shutter unit. The fast shutter is based on a Galvano motor inside a vacuum cube. On the Galvano motor head, two special custom-made Densimet pieces (rhombus-shaped) are mounted. A total maximum rotation of 40° is sufficient to either block or fully open an aperture of 2.5 mm. The open and closing time is 2.2 ms for a total travel range of 40°. Depending on the actual beam size the attenuation of the full beam is faster than 2.2 ms. A feedback analog signal from the Galvano motor is fed into the control system to wait for the shutter to be opened or closed completely. Due to the small aperture of this unit, the vacuum cube with the shutter inside can be vertically and horizontally adjusted via stepper motors. Further downstream a beam intensity monitor is installed. A windowless photodiode (Hamamatsu, S1337-21) inside a vacuum cube collects light and fluorescence originating from a nitro­gen-doped CVD diamond sheet (50 µm thickness) that can be moved at 45° into the beam axis. As a cross-check of the beam intensity, an additional ionization chamber filled with nitro­gen (1 bar) is installed in front of the guard slit (JJ X-ray).

For the sample environments either from the users or the beamline equipment an optical breadboard table (700 mm × 800 mm) is installed at the nominal sample position. This table can move 250 kg by 300 mm up or down. Downstream of the sample table, the in-house-constructed/built SAXS/WAXS instrument is permanently installed.

#### 2.4.1. SAXS/WAXS instrument

**Figure 4:**
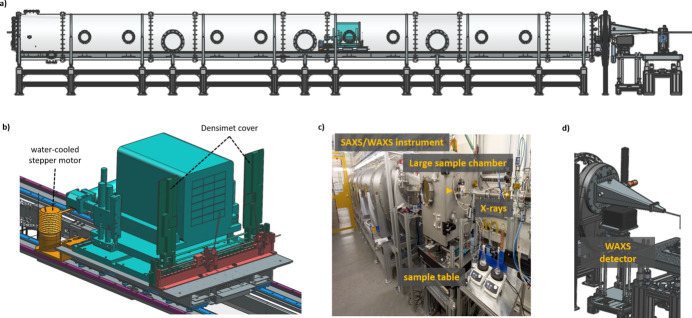
(*a*) Side view of a 3D CAD-model of the SAXS/WAXS instrument. (*b*) The SAXS detector mounting base with additional beamstops and protective cover. (*c*) Photograph of the system in the experimental hutch. (*d*) WAXS detector assembly.

Figure 4 ▸ shows a 3D CAD-model of the realized SAXS/WAXS instrument. The SAXS instrument is composed of a 13 m-long and 1 m-diameter stainless steel tube (304L) with inside motorized translation stages for the SAXS detector and beamstops. The wall thickness is 15 mm to ensure low vibration and no deformation while moving the SAXS detector system inside the tube system. The different vacuum tube sections (1 m- and 2 m-long) have been manufactured by Pfeiffer Vacuum including support brackets inside for mounting the inside translation system. The translation system inside has been built internally at DESY. The vacuum tube system (operating pressure: 7.5 × 10^−4^ mbar) is supported underneath by an aluminium-profile frame system from the company Rose+Krieger. Inside the tube, a rail system allows continuous movement of the SAXS detector system from 1.9 m to 13.0 m to the sample position. The guiding rails are from the company HEPCO Motion and are optimized for vacuum applications. The motorized motion is realized by two parallel rack and pinion drive systems in combination with electronically synchronized stepper motors. In Fig. 4 ▸(*b*) the water-cooled stepper motors can be seen behind the detector system (the yellow items).

The SAXS detector, an Eiger2 X 9M (Dectris), is mounted on a secondary translation stage for off-centering the detector horizontally and vertically, such that the direct beam can hit the detector either in the center or the lower left/right corner. By this option, the accessible *q*-range for isotropic scattering samples can be nearly doubled.

In front of the SAXS detector, two large 2 mm-thick Densimet plates are installed as a protective cover during X-ray beam alignment tasks [dark green items in Fig. 4 ▸(*b*)]. These plates are motorized with individually driven stepper motors. Directly in front of this protecting cover one beam-stop unit with two separate motorized beam-stops is installed. There is an option to install two more identical beam-stop units with different beam-stops in front of it. Currently, a 4.5 mm and a 6.0 mm round active beam-stop are used. These beam-stops are based on a Densimet cylinder with a Ce:Gd_3_Al_2_Ga_3_O_12_ crystal inside (Advatech UK Limited). Inside the metal supporting stick a 600 µm glass fiber is used to guide the produced visible light (wavelength: 530 nm) from the crystal to the outside of the vacuum vessel, where the light signal will be converted into an electrical voltage signal (Femto: 200 MHz Variable Gain Photoreceiver Series OE-300). The intensity of the signal is proportional to the X-ray beam flux. The measured beam intensity with the active beamstop is calibrated against a calibrated ionization chamber filled with nitro­gen gas at 1 bar as a function of X-ray energy and flux.

In front of the SAXS tube, a DN500 high-vacuum pendulum isolation valve (VAT Vakuumventile AG) is installed to separate the SAXS vacuum system from the sample environment vacuum system while mounting the vacuum sample setup or/and changing the samples within this vacuum sample chamber. From the nominal sample position to the large valve, a customized vacuum cone is installed. Below this cone, the Eiger2 X 4M-DESY detector from Dectris is placed as the WAXS detector. The shape of this detector, especially the top housing shape, matches the vacuum cone shape to reduce the gap between the SAXS and WAXS signal. Another modification of the customized Eiger2 X 4M-DESY is that the first active pixel row is just 7 mm below the upper housing of the detector, which further reduced the gap between the SAXS and WAXS signal. Otherwise, the specification of the Eiger2 X 4M-DESY are the same as the conventional Eiger2 X 4M.

A summary of the key specification of the SAXS/WAXS instrument is given in Table 4 ▸, including the accessible *q*-range for the SAXS and WAXS detector at 12.4 keV. The *q*-range depends strongly on the X-ray energy as well as on the sample environment. The values reported in Table 4 ▸ are the default parameters.

#### 2.4.2. Sample environment

**Figure 5:**
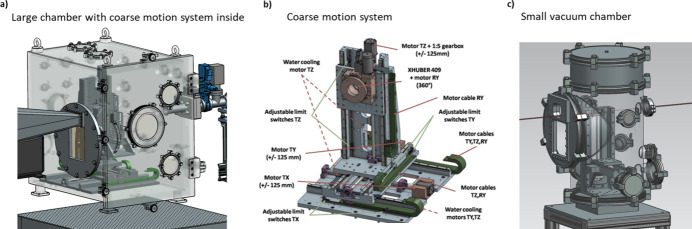
Sample environments. (*a*) Large chamber with a coarse motion system inside (height × depth × width: 750 mm × 750 mm × 550 mm). (*b*) Detailed view of the coarse motion system. (*c*) Small vacuum chamber with limited motions inside (inner diameter 250 mm). The small chamber can be directly connected to the SAXS instrument without a SAXS window. The WAXS window is a carbon fiber ep­oxy sheet. The large chamber is normally separated from the SAXS instrument by a Kapton window.

The beamline has been designed such that the sample environment area is a free space for mounting user equipment or special environments from the beamline. At the sample position area, an optical breadboard table (700 mm × 800 mm) is permanently installed. This table can lift roughly 250 kg. The user equipment can be placed on this table and adjusted in height to match the X-ray beam height. At the beamline, several environments are available, such as a large and a small chamber housing different setups. The large chamber can be filled with He gas or evacuated to 1 × 10^−4^ mbar. This chamber houses a coarse motion system with four translation degrees of freedom. In Fig. 5 ▸(*a*) the chamber is shown and Fig. 5 ▸(*b*) shows a detailed 3D CAD-model of the coarse motion system. This setup is mainly used for the SAXS/WAXS tensor tomography experiments in combination with an additional fine motion scanning system from SmarAct. For standard ASAXS/AWAXS experiments, a small vacuum chamber [Fig. 5(*c*) ▸] is available that has two translation degrees of freedom. This small chamber can be directly connected to the SAXS/WAXS instrument without any extra window. Therefore, a very low scattering background can be achieved with this setup.

To mount and align small user equipment that cannot be placed inside a vacuum, a second identical coarse motion system is available that can be operated in air and can be mounted on the optical breadboard table. This coarse-motion system can handle equipment with a maximum of 5 kg weight. Besides the sample environments, the beamline develops user-accessible equipment for *in situ* and *operando* experiments. The equipment often used at the beamline includes a capillary heater that can reach a temperature up to 1000°C with a gas flow connection, a setup with five individually controlled heaters up to a temperature of 500°C, a capillary flow-through cell connected to a syringe pump system, and a tensile stage with forces from 0.01 N to 600 N.

#### 2.4.3. Control system

The control of the beamline is based on Tango Device Server (TANGO, 2023 ▸) and Sardana (Sardana, 2023 ▸). Respectively, a command line interface called ‘spock’ is used to communicate with Sardana or we can use custom graphical user interfaces written in Python using the PyTango (PyTango, 2023 ▸) package to access the Tango devices and the Sardana macro server.

**Figure 6:**
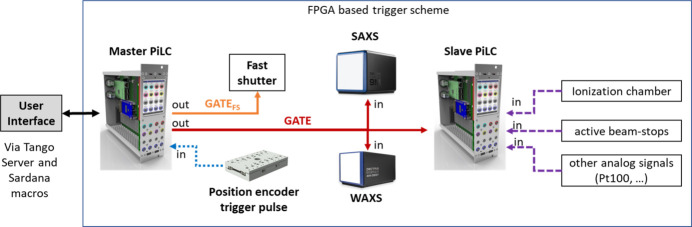
Simplified overview of the triggering scheme based on preprogrammed FPGAs.

In Fig. 6 ▸ an overview of the acquisition system is shown. The core of this system is two PiLCs (Raspberry Pi Logic Controller). The basic components of a PiLC are an embedded PC, an FPGA chip and IO Cards interfaces for digital and analog signals. During operation, the embedded PC loads the firmware, which has been developed for a specific application, into the FPGA, activates it, and provides access to the FPGA registers. This device has been developed in-house at DESY. The user interacts with these devices via the Tango server and Sardana macros. The master PiLC generates signal GATES that can be used to trigger the exposure of different devices such as the exposure of the SAXS and WAXS detector or the acquisition of the slave PiLC. The slave PiLC records analog signals for the time the input GATE is in the high state. Depending on the requirements of the SAXS/WAXS experiments, different trigger control modes can be realized at the beamline.

**Figure 7:**
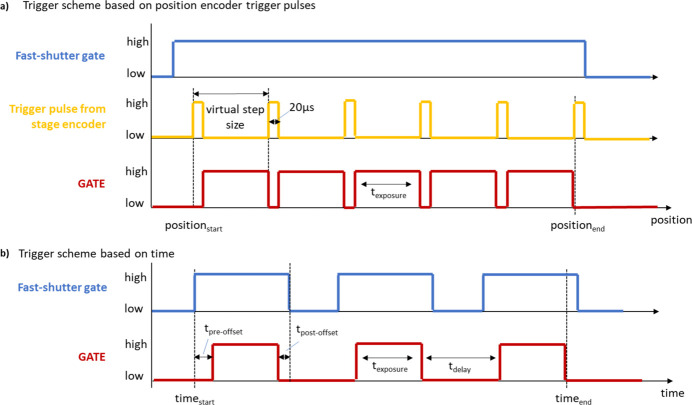
Trigger schemes implemented at the beamline. (*a*) Trigger scheme based on trigger pulses from a position encoder electronic. (*b*) Trigger scheme based on time.

For very demanding SAXS/WAXS tensor tomography kinds of experiment, an on-the-fly data trigger scheme has been implemented using special trigger pulses from the encoder position readout of the scanning linear stages from the company SmarAct. These trigger pulses are fed into the master PiLC. The master PiLC puts the gate signal to a high state once the trigger pulse from the stage has a falling edge. Until the next position trigger pulse arrives, the gate stays in the high state and the detectors and other devices are counting. Once the next trigger pulse arrives and has a rising edge, the master PiLC switches the gate signal to a low state and the acquisition is stopped for all devices. Figure 7 ▸(*a*) shows the trigger and produced gate signal for this measurement scheme. The user can adapt the exposure time for a virtual step of the continuous moving linear stage. The length of the trigger pulse from the encoder is fixed at 20 µs. Typically, the exposure time of a virtual step is of the order of 20 ms. That means with a continuous motion mode operation data are collected for more than 99.9% of the time. With this data acquisition mode, the fast shutter opens at the beginning of the continuous motion and closes after the whole motion is finished.

In the case of *in situ* and anomalous SAXS/WAXS experiments, the triggering is time-based. In Fig. 7 ▸(*b*) an overview of the gate signals for this mode is shown. The user can define the length of the main exposure gate signal in the range 4 ms to 600 s as well as the number of repetitions. This mode allows the definition of a delay time between two exposures. This delay time can be adjusted from 20 µs to 600 s with a 1 µs resolution. If the delay time is smaller than 5 ms, the fast shutter will stay in the open position for all exposures and waiting times. In cases where the delay time is larger than 5 ms, the fast shutter will be opened and closed for each exposure. To guarantee that the fast shutter is fully opened before the acquisition gate signal is set high, a second fast shutter control gate is produced by the master PiLC with some pre- and post-offset times. This means the fast shutter starts opening roughly 2.5 ms before the main gate signal and starts closing roughly 1.5 ms after the main gate signal is finished. These two offset times can be adjusted as well.

## 3. Examples

### 3.1. ASAXS study of PVP-coated dried Au nanoparticles

To stabilize and prevent agglomeration of nanoparticles in solutions such as water, special additives are added to the synthesis process (Studart *et al.*, 2007 ▸; Liu *et al.*, 2022 ▸; Koczkur *et al.*, 2015 ▸). These surfactants form a protecting layer around the primary nanoobject. Polyvinyl­pyrrolidone (PVP) is one often-used additive that can act as a surface stabilizer, growth modifier, nanoparticle dispersant and reducing agent (Koczkur *et al.*, 2015 ▸). PVP prevents aggregation of nanoparticles via repulsive forces that arise from the hydro­phobic carbon chains that extend into the solvents and interact with each other [steric hindrance effect (Quesada-Pérez *et al.*, 2021 ▸)]. Due to the organic nature of those surfactant shells, normally neutron scattering experiments are more suitable due to the higher scattering contrast for neutrons. In many cases, the scattering contrast of the organic shell is too weak to be detectable by X-ray scattering experiments. Recently a novel approach has been realized by combining small-angle neutron scattering (SANS) with SAXS at one instrument (Metwalli *et al.*, 2020 ▸). However, the time resolution of SANS experiments is much lower compared with SAXS and the sample transmission has to be optimized for both X-rays and neutrons at the same time. Furthermore, other approaches could be used to vary the scattering contrast of the organic shell. This can be achieved by either using different solvents or exploring the anomalous scattering effect in X-ray scattering experiments (Sakou *et al.*, 2013 ▸; Haas *et al.*, 2010 ▸; Vainio *et al.*, 2012 ▸; Hoell *et al.*, 2009 ▸).

**Figure 8:**
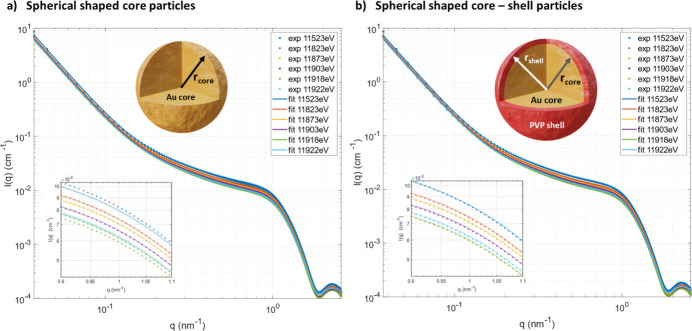
Experimental and modeled SAXS intensities using (*a*) a polydisperse spherical-shaped core particle model and (*b*) a polydisperse spherical-shaped core-shell particle model.

The question is: is it possible to detect an organic protecting shell around an inorganic metallic nanoparticle? To answer this question, we investigated a 5 nm-diameter gold nanosphere covered by a PVP layer from the company NanoXact using ASAXS at the Au-*L*_3_ X-ray absorption edge at the new beamline. The symbols in Fig. 8 ▸ show the measured absolute differential scattering cross-section of the sample measured at six different X-ray energies below the Au-*L*_3_ X-ray absorption edge. A clear energy dependency of the intensity can be seen. The scattering curves have been normalized by the incoming photon flux and the sample transmission. The normalized intensity was subtracted from the normalized scattering intensity of an empty sample container as background. Finally, the intensity was normalized to absolute differential scattering units in cm^−1^ using the secondary standard sample glassy carbon (Zhang *et al.*, 2010 ▸; Dreiss *et al.*, 2006 ▸). Based on transmission electron microscope (TEM) analysis of the NanoXact company the particles can be assumed to be spheric with a certain size distribution. To check whether it is possible to detect the PVP shell, the scattering curves were modeled with two different models: (1) spherical particles with a log-normal size distribution and a structure factor to account for particle interaction, and (2) spherical core-shell particles with a log-normal size distribution and a structure factor. Model 1 corresponds to the scenario that the PVP shell is not detectable by X-ray scattering and model 2 corresponds to the case that the PVP shell is detectable. The solid lines in Fig. 8 ▸ show the modeled intensities. The quality of both non-linear regressions is very good. The quality is slightly better for the core-shell model [inset plot Fig. 8 ▸(*b*)] compared with that of the spherical particle model [inset plot Fig. 8 ▸(*a*)]. A detailed description of the model equations and the fitting procedure is given in the supporting information. The key advantage of the ASAXS modeling is that it is possible to perform a combined non-linear regression of all measured scattering curves at different energies. Because some model parameters such as radius, number density, *etc*. have to be energy independent, the effective number of model parameters per scattering curve is reduced compared with a single curve fitting. In the case of the spherical model, the number of model parameters per curve is reduced from ten parameters for the single fitting to effectively 4.2 parameters for the combined fit of six curves. In the case of the core-shell model, the number is reduced from 12 parameters to effectively 4.5.

**Figure 9:**
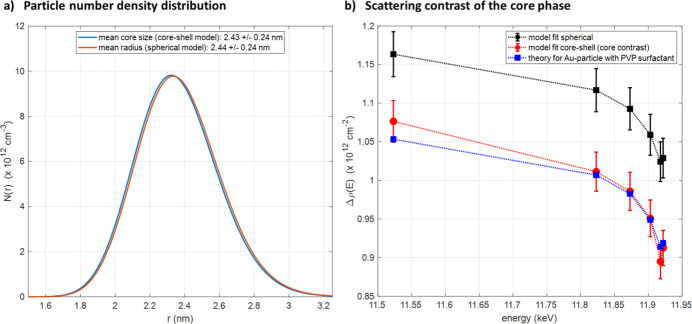
(*a*) Particle number density of both models. The mean radius is almost equal to 2.44 nm. (*b*) The energy-dependent scattering contrast of both models compared with the theoretical contrast for an Au core against a PVP shell. The symbols are the obtained values from the non-linear regression and the lines are linear interpolations of them for guidance only.

Figure 9 ▸ shows the corresponding number density distribution as a function of particle radius. Both models give a very similar distribution with a mean value of the radius of 2.44 nm and a polydispersity of 9.8%. These values are in good agreement with the analysis from NanoXact which reports a radius of 2.6 nm and a polydispersity of 12% based on the TEM analysis. To answer the question about the detectability of the PVP shell, the energy-dependent scattering contrast of the core has to be analyzed in more detail. Figure 9 ▸(*b*) shows the obtained scattering contrast as a function of X-ray energy for the pure spherical particle (black symbols) and the core of the core-shell particle (red symbols). The contrast values are higher for the spherical particle model compared with the core-shell model. In addition, the theoretical scattering contrast for gold against PVP as a function of X-ray energy was calculated and plotted in Fig. 9 ▸(*b*) as a reference (blue symbols). The agreement of the theoretical values is much better for the core-shell model compared with the pure spherical model. Therefore, the core-shell model is the better model assumption for modeling the measured SAXS curves. In conclusion, the ASAXS technique allows the detection of organic surfactant layers around metallic nanoparticles. This approach enables further X-ray studies on the effect of the protection layers on the performance of functionalized nanoparticles using *in situ* and *operando* X-ray experiments such as *in situ* ASAXS with a time resolution of a few seconds.

### 3.2. SAXS-CT of a phantom sample

**Figure 10:**
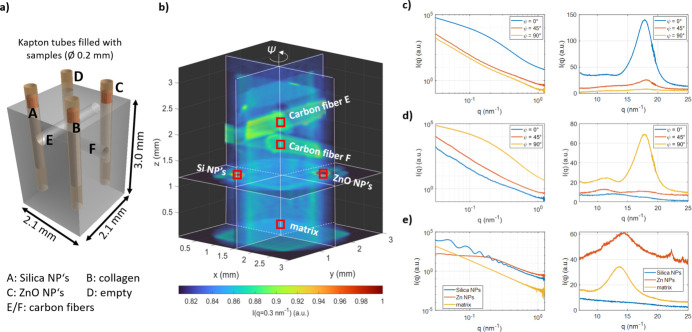
(*a*) Sketch of the phantom sample assembly. The Kapton tubes are filled with different samples (A = silica nanoparticles, B = collagen lower part only, C = ZnO nanoparticles, D = empty, E and F = carbon fibers). (*b*) SAXS-CT reconstruction intensities at *q* = 0.3 nm^−1^. Reconstructed SAXS and WAXS intensities of certain voxels: (*c*) carbon fiber location E as a function of view angle ψ, (*d*) carbon fiber location F as a function of view angle. (*e*) Si nanoparticles, ZnO nanoparticles and solid water matrix locations.

To commission and optimize the measurement scheme and processing pipeline of the SAXS/WAXS tensor tomography experiments a phantom sample has been prepared and a full SAXS/WAXS computed tomography dataset has been collected at the SAXSMAT beamline. A detailed description and full data analysis will be published elsewhere. Here, only a few reconstructions will be discussed briefly. In Fig. 10 ▸(*a*) a sketch of the phantom sample is shown. The different samples are placed in a solid water cube (2.1 mm × 2.1 mm × 3.0 mm) with 0.3 mm-diameter holes. Hereby, each sample has been placed in a 0.2 mm-diameter Kapton tube with 0.025 mm wall thickness. Solid water is a solid polymer that has the same electron density as liquid water. Different samples have been placed inside the phantom: amorphous silica nanospheres with 120 nm diameter (Sigma Aldrich), crystalline ZnO nanoparticles with ∼20 nm radius, collagen fibers and carbon fibers. The collection was chosen such that each of them scatters at different *q*-values and has different scattering power or even orientations.

The SAXS/WAXS computed tomography dataset has been collected with the following experimental settings: X-ray energy = 11 keV, beam size = 25 µm × 10 µm (horizontal × vertical), virtual step size of the vertical and horizontal directions = 30 µm, number of virtual translation steps in the horizontal direction = 110 (continuous scan direction), number of translation steps in the vertical direction = 115, number of rotation steps around the vertical axis = 61 (3.0° step size) and exposure time per virtual point = 50 ms. In total 771650 SAXS and 771650 WAXS patterns have been collected in ∼13 h. The total exposure time is 10.7 h with an additional 2.3 h of overhead due to the step-wise motion of the rotation and height motion as well as the arming time for the trigger and detector units. The overhead factor of ∼1.2 is small compared with a fully step wise scan with an overhead of 3.2. The dataset has been processed with an in-house-developed processing pipeline, which will be described in a separate publication. In Fig. 10 ▸(*b*) the reconstructed 3D tomogram of the SAXS intensity at *q* = 0.3 nm^−1^ is shown in a 3D sliced view model. In the upper part of the volume, two crossing horizontal channels can be seen with higher intensity. These are carbon fibers. In the horizontal cutting plane at *z* = 1.2 mm, two round spots with higher intensities are visible. The spot at location (1.0, 1.0, 1.2) belongs to channel A which was filled with silica nanoparticles. The second spot at (2.0, 2, 0, 1.2) mm belongs to channel C which was filled with ZnO nanoparticles.

A kind of reverse analysis can be carried out at any voxel of interest to retrieve the SAXS or/and WAXS scattering curve as a function of the scattering vector for that voxel. This scattering curve represents the scattering curve of only that particular voxel in the X-ray beam. Figures 10 ▸(*c*)–10(*e*) show the obtained scattering curves for selected voxels. In Figs. 10 ▸(*c*) and 10 ▸(*d*) the curves for the two different fiber locations are shown as a function of the view angle ψ. The view angle is important for carbon fibers because they are highly oriented. For the fiber located in channel E [Fig. 10 ▸(*c*)] the SAXS and WAXS intensity is highest for the view angle ψ = 0° and lowest for ψ = 90°. The broad peak around 18 nm^−1^ corresponds to carbon fiber. For the carbon fiber located in channel F, it is the opposite behavior because the channel is rotated by 90°. In Fig. 10 ▸(*e*) the retrieved scattering curves for the location of the silica nanoparticles, ZnO nanoparticles and solid water matrix are shown. The SAXS curves of the silica nanoparticles show mainly the spherical form factor with a very narrow size distribution while the scattering curve of the ZnO particles represents particles with a much larger polydispersity as expected. The corresponding WAXS curves are also shown in Fig. 10 ▸(*e*). At *q* = 22.5 and 24.0 nm^−1^ two weak peaks are present in the ZnO location, indicating the presence of crystalline ZnO nanoparticles. On the other hand, no diffraction peaks were observed in the accessible region for the silica nanoparticles, indicating that the Si nanoparticles are amorphous rather than crystalline.

This example shows nicely the quality of the implemented SAXS/WAXS imaging setup at the new SAXSMAT beamline. A detailed description of the performance and data processing pipeline for the SAXS/WAXS tomography experiments at the SAXSMAT beamline will be published in a separate paper including a full analysis of the phantom sample concerning particle distribution, fiber orientation and degree of alignment.

## 4. Summary

The new SAXSMAT beamline P62 at PETRA III is a versatile SAXS/WAXS instrument for applications of both hard and soft condensed matter research in the fields of chemistry, materials and life sciences, physics and related disciplines. This manuscript describes the beamline’s layout in terms of optics configuration and performance as well as the SAXS/WAXS instrumentation. The design parameters of the beamline were successfully achieved. In addition, two selected examples were shown to highlight the capability using the advanced techniques of anomalous scattering and computed tensor tomography that was realized at the beamline.

## 5. Related literature

The following references, not cited in the main body of the paper, have been cited in the supporting information: Baxter (1970 ▸); Glatter & Kratky (1982 ▸); Pedersen (1994 ▸).

## Supplementary Material

Sections S1 to S2.4. DOI: 10.1107/S1600577523008603/ay5619sup1.pdf

## Figures and Tables

**Table 1:** Specifications of the beamline optics

Parameter	Specification
Source	Undulator (U32)
Monochromator	Cryo-cooled DCM [Si(111) and Si(311)]
Energy range (keV)	3.5–35.0
Mirror stripes	B_4_C, Rh, Pt
Flux (photons s^−1^ @ 12 keV at 120 mA)	1.2 × 10^13^
Focus size at sample (H × V) (µm)	10 × 10 to 500 × 500

**Table 2:** Specifications of the beamline photon source

Parameter	Specification
Device length (m)	2.0
Period length (mm)	31.4
Peak field *B*_0_ (T)	0.91
Number of periods	61
Minimum magnetic gap (mm)	9.5
Deflection parameter *K*_max_	2.7
Energy of first harmonic (keV)	2.4

**Table 3:** Summary of the DCM specifications

	Travel range	Repeatability	Minimum step resolution
C1pitch (Bragg axis)	−3.0°–40.0°	<0.20 µrad	<0.10 µrad
C1roll	±0.5°	5.0 µrad	1.5 µrad
C2pitch	±0.5°	5.0 µrad	1.5 µrad
C2pitch-piezo	±150 µrad	0.1 µrad	0.015 µrad
C2roll	±0.5°	5.0 µrad	1.5 µrad
C2x	10–240 mm	2.5 µm	0.6 µm
C2z	10–30 mm	2.5 µm	0.6 µm

**Table 4:** Specifications of the SAXS/WAXS instrument

Parameter	Specification
X-ray energy range (keV)	3.5–35.0
Energy resolution	Δ*E*/*E* = 1.4 × 10^−4^
Sample to SAXS detector distance (m)	1.9–13.0
Sample to WAXS detector distance (m)	0.2–0.5
Flux (photons s^−1^ at 120 mA)	9.1 × 10^12^ @ 7 keV
	1.2 × 10^13^ @ 12 keV
	5.5 × 10^12^ @ 20 keV
	1.5 × 10^12^ @ 35 keV
Focus size at sample (H × V) (µm)	10 × 10 to 500 × 500
Beam stop diameter (mm)	4.5 and 6.0
*q*-range SAXS at 12.4 keV	0.04–4.5 nm^−1^ (detector centered)
	0.04–6.0 nm^−1^ (detector off-centered)
*q*-range WAXS at 12.4 keV	7.0–38.0 nm^−1^
