# Supplementary material for: The new small-angle X-ray scattering beamline for materials research at PETRA III: SAXSMAT beamline P62
Source: J Synchrotron Radiat. 2023 Oct 20;30(Pt 6):1156–67. doi: 10.1107/S1600577523008603 (PMC10624033; doi:10.1107/S1600577523008603)
Supplement: Supplementary file 1 [file s-30-01156-sup1.pdf]

JOURNAL OF SYNCHROTRON RADIATION

Volume 31 (2024)

Supporting information for article:

The new small-angle X-ray scattering beamline for materials research at PETRA III: SAXSMAT beamline P62

S. Haas, X. Sun, A. L. Conceicao, J. Horbach and S. Pfeffer

S1. Additional figures

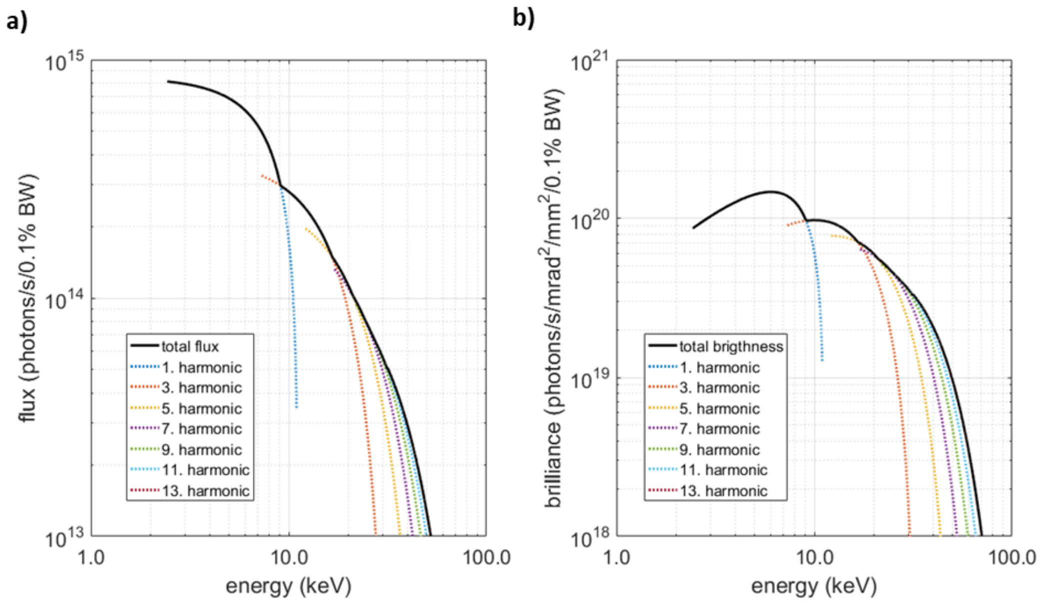

Figure S1 Flux and brilliance of the U32 undulator of the SAXSMAT P62 beamline.

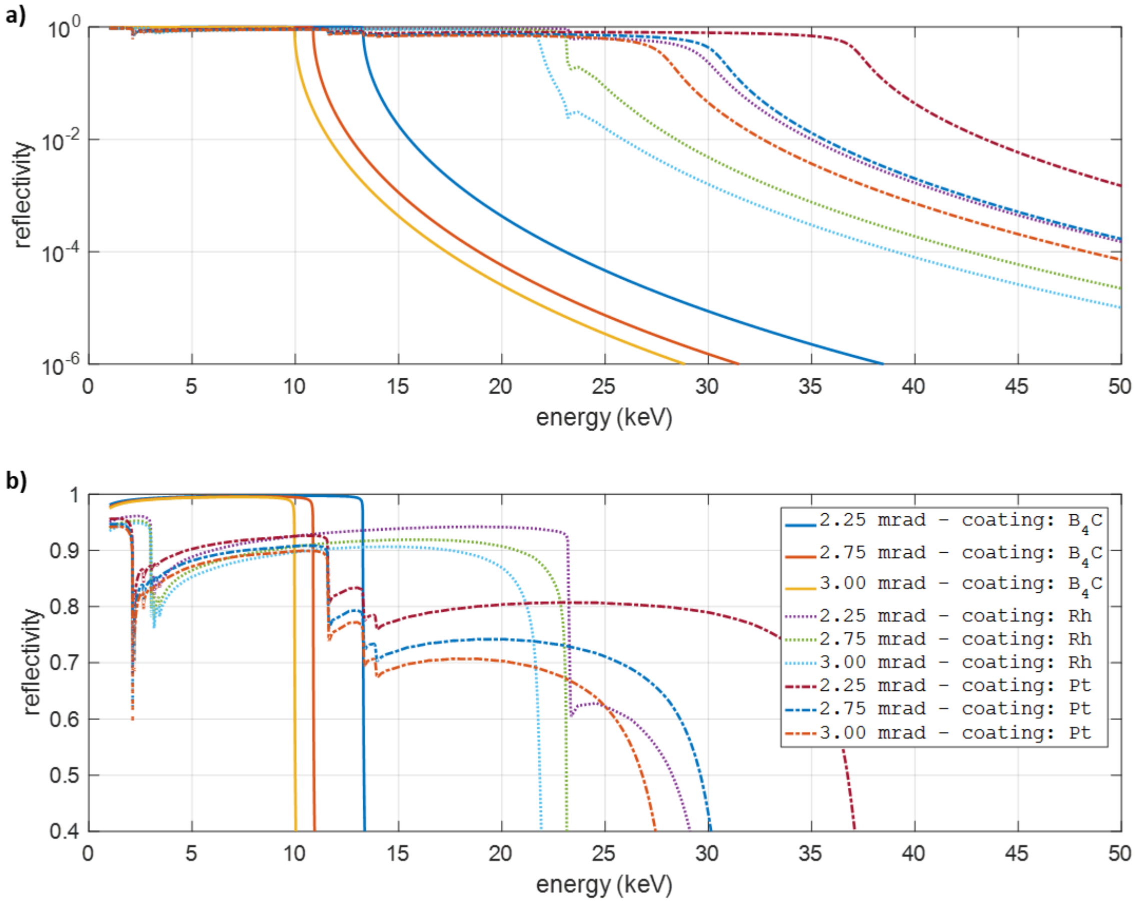

Figure S2 Calculated reflectivity curves of the double mirror system as a function of X-ray energy, pitch angle, and coating.

S2. ASAXS study of PVP-coated dried Au nanoparticles

S2.1. Spherical nanoparticle ASAXS model

The intensity of spherical nanoparticles with a lognormal size distribution and a structure factor in the local monodisperse approximation is given by (Glatter & Kratky, 1982; Pedersen, 1994):

$$I(q,E) = N\int_0^\infty P(r,\mu,\sigma)F_s(q,r,\rho(E))^2 S(q,r,rhs,\tau,\nu)dr + \beta_1(E)q^{-\alpha} + \beta_0(E).$$

N is the number density of particles in particles per cm³, μ and σ are the size distribution $P(r,\mu,\sigma)$ parameters (mean and polydispersity).

$F_s(q,r,\rho(E))$ is the form factor of spherical particles given by:

$$F_s\big(q,r,\rho(E)\big) = \frac{4}{3}\pi r^3\rho(E)3\frac{\sin(qr)-qr\cos(qr)}{(qr)^3}.$$

$\rho(E)$ is the energy E dependent scattering contrast of the particle phase against the solvent.

As structure factor $S(q,r,rhs,\tau,\nu)$ the sticky hard sphere model has been used (Baxter, 1970). This model includes three additional model parameters: rhs is the repulsive radius, τ the stickiness parameter characterizing the adhesive strength, and ν is the local volume fraction of particles.

The scattering background is modeled by the last two terms including two energy-dependent parameters $\beta_0(E)$ and $\beta_1(E)$ and one energy-independent parameter α that should be between 3 and 4.

S2.2. Spherical core-shell nanoparticle ASAXS model

The intensity of spherical core-shell nanoparticles with a lognormal size distribution and a structure factor in the local monodisperse approximation is given by (Glatter & Kratky, 1982; Pedersen, 1994):

$$I(q,E) = N\int_0^\infty P(r,\mu,\sigma)F_{cs}(q,r,d,\rho_{core}(E),\rho_{shell})^2 S(q,r,rhs,\tau,\nu)dr + \beta_1(E)q^{-\alpha} + \beta_0(E).$$

N is the number density of particles in particles per cm³, μ and σ are the size distribution $P(r,\mu,\sigma)$ parameters (mean and polydispersity).

$F_{cs}(q,r,d,\rho_{core}(E),\rho_{shell})$ is the form factor of spherical core-shell particles given by:

$$F_{cs}(q,r,d,\rho_{core}(E),\rho_{shell}) = F_s(q,r+d,\rho_{shell}) - F_s(q,r,\rho_{shell}-\rho_{core}(E)) .$$

$\rho_{core}(E)$ is the energy E dependent scattering contrast of the particle core phase against the solvent and ρ_{shell} is the scattering contrast of the particle shell against the solvent phase, which can be assumed to be energy independent. In the present case water. d is the shell thickness.

As structure factor $S(q,r,rhs,\tau,\nu)$ the sticky hard sphere model has been used (Baxter, 1970). This model includes three additional model parameters: rhs is the repulsive radius, τ the stickiness parameter characterizing the adhesive strength, and ν is the local volume fraction of particles.

The scattering background is modeled by the last two terms including two energy-dependent parameters $\beta_0(E)$ and $\beta_1(E)$ and one energy-independent parameter α that should be between 3 and 4.

S2.3. Summary of all fit parameters spherical model

parameter	E=11523eV	E=11823eV	E=11873eV	E=11903eV	E=11918eV	E=11922eV
$\boldsymbol{\alpha}$	3.52 ± 0.01					
$\boldsymbol{\beta_0}$	0.0028 ± 0.0001	0.0022 ± 0.0001	0.0021 ± 0.0001	0.0021 ± 0.0001	0.0021 ± 0.0001	0.0036 ± 0.0001
$\boldsymbol{\beta_1}$	0.0071 ± 0.0003	0.0064 ± 0.0002	0.0062 ± 0.0001	0.0061 ± 0.0001	0.0057 ± 0.0001	0.0058 ± 0.0001
$\boldsymbol{N}$	5.734e-4 ± 5.97e-7					
$\boldsymbol{\mu}$	2.333 ± 0.001					
$\boldsymbol{\sigma}$	0.0995 ± 0.0004					
$\boldsymbol{\rho}$	1.163 ± 0.002	1.117 ± 0.003	1.093 ± 0.002	1.059 ± 0.001	1.024 ± 0.003	1.029 ± 0.002
$\boldsymbol{rhs}$	3.628 ± 0.005					
$\boldsymbol{\tau}$	0.142 ± 0.002					
$\boldsymbol{\nu}$	0.069 ± 0.001					

The weighted R-value of the regression is: 0.0214425.

S2.4. Summary of all fit parameters spherical core-shell model

parameter	E=11523eV	E=11823eV	E=11873eV	E=11903eV	E=11918eV	E=11922eV
$\boldsymbol{\alpha}$	3.52 ± 0.01					
$\boldsymbol{\beta_0}$	0.0021 ± 0.0002	0.0020 ± 0.0001	0.0020 ± 0.0001	0.0021 ± 0.0001	0.0028 ± 0.0001	0.0038 ± 0.0001
$\boldsymbol{\beta_1}$	0.0070 ± 0.0002	0.0065 ± 0.0001	0.0063 ± 0.0001	0.0061 ± 0.0001	0.0058 ± 0.0001	0.0059 ± 0.0001
$\boldsymbol{N}$	5.734e-4 ± 5.97e-7					
$\boldsymbol{\mu}$	2.323 ± 0.001					
$\boldsymbol{\sigma}$	0.0998 ± 0.0004					
$\boldsymbol{d}$	0.103 ± 0.012					
$\boldsymbol{\rho_{core}}$	1.1864 ± 0.0002	1.1213 ± 0.0001	1.0958 ± 0.0001	1.0608 ± 0.0001	1.0049 ± 0.0001	1.0225 ± 0.0001
$\boldsymbol{\rho_{shell}}$	0.11002 ± 0.00012					
$\boldsymbol{rhs}$	3.626 ± 0.005					
$\boldsymbol{\tau}$	0.143 ± 0.002					
$\boldsymbol{\nu}$	0.070 ± 0.001					

The weighted R-value of the regression is: 0.0132957.
